# Encapsulation of S/SWNT with PANI Web for Enhanced Rate and Cycle Performance in Lithium Sulfur Batteries

**DOI:** 10.1038/srep08946

**Published:** 2015-03-10

**Authors:** Joo Hyun Kim, Kun Fu, Junghyun Choi, Kichun Kil, Jeonghyun Kim, Xiaogang Han, Liangbing Hu, Ungyu Paik

**Affiliations:** 1 Department of Energy Engineering, Hanyang University, Seoul 133-791, South Korea; 2 Department of Materials Science and Engineering, Hanyang University, Seoul 133-791, South Korea; 3 Department of Materials Science and Engineering, University of Maryland, College Park, Maryland 20742, United States

## Abstract

Lithium-sulfur batteries show great potential to compete with lithium-ion batteries due to the fact that sulfur can deliver a high theoretical capacity of 1672 mAh/g and a high theoretical energy density of 2500 Wh/kg. But it has several problems to be solved in order to achieve high sulfur utilization with high Coulombic efficiency and long cycle life of Li-S batteries. These problems are mainly caused by the dissoluble polysulfide species, which are a series of complex reduced sulfur products, associating with shuttle effect between electrodes as well as side reactions on lithium metal anode. To alleviate these challenges, we developed a sulfur-carbon nanotube (S/SWNT) composite coated with polyaniline (PANI) polymer as polysulfide block to achieve high sulfur utilization, high Coulombic efficiency, and long cycle life. The PANI coated S/SWNT composite showed a superior specific capacity of 1011 mAh/g over 100 cycles and a good rate retention, demonstrating the synergic contribution of porous carbon and conducting polymer protection to address challenges underlying sulfur cathode.

To meet with high energy storage demanding, significant efforts have been placed to explore new types of battery chemistries and configurations that have a combination of high energy density, improved safety, and reduced cost^1^,^2^,^3^. Lithium-sulfur (Li-S) batteries have attracted great attentions over the past few years due to their appealing performance that can potentially deliver a theoretical energy density of 2500 Wh/kg^4^,^5^. Sulfur characterizes not only a high theoretical capacity of 1672 mAh/g with average working voltage at around 2.1 V (V vs. Li/Li^+^), but also the natural abundance, low toxicity, and reasonable cost, which makes it an ideal cathode material for high-energy density lithium batteries.

However, main challenges on how to achieve high sulfur utilization with high Coulombic efficiency and long cycle life are remained to get superior Li-S batteries performance^6^,^7^,^8^. These challenges are mostly associated with the shuttle effect of dissoluble polysulfide species that cause active materials loss and side reactions on lithium metal anode. It will directly results in capacity fading, coulombic efficiency decrease, lithium metal corrosion, and dendrite formation. To suppress the shuttle effect, it is necessary to completely preserve sulfur and polysulfides locally in cathode area. Thus, many studies have been conducted to design all kinds of sulfur-based nanocomposites, especially focusing on sulfur-carbon materials that have sulfur anchored inside^8^. Representative sulfur-carbon structure is reported by Nazar's group on a highly ordered mesoporous carbon-based sulfur composite^9^. The mesoporous structure not only created high surface area for a controlled sulfur loading, but also provided anchoring sites to capture polysulfides, enabling the stabilized capacity over 1000 mAh/g. Different types of carbon materials including graphene, carbon nanotubes, and carbon nanofibers have intensively motivated researchers to design sulfur-carbon composites owing to their good electronic conductivity^10^,^11^,^12^,^13^,^14^,^15^,^16^,^17^. The improved electrochemical performance of sulfur cathode contributed from highly porous carbon structures expresses a promising sulfur-based composite development direction. To further block the leakage of polysulfides from sulfur-carbon composites, typically two designs have been employed: (a) inserting an interlayer in between cathode and separator for intercepting the diffusing polysulfides^18^,^19^,^20^, and (b) applying a thin layer of polymer coating to protect sulfur-carbon composite for blocking the out-diffusion of polysulfides^21^,^22^,^23^,^24^. Compared to interlayers, the thin polymer coating will not increase the mass burden of total battery configurations, which will not compromise the energy density of batteries. Therefore, sulfur-carbon composite with a protection layer arouses a great interest of research to study the synergic contribution of porous carbon and polymer coatings and to find a facile but effective way of utilizing sulfur while suppressing the out-diffused polysulfides locally, in order to address the challenges in terms of polysulfides dissolution in cathode and migration between electrodes.

In this work, we present a sulfur-carbon composite coated with a conducting polymer as polysulfide block to increase the Li-S batteries electrochemical performance. The sulfur-carbon composite consists of a free-standing single-walled carbon nanotube (SWNT) film loaded with thermo-infused sulfur. The free-standing SWNT film is an ideal carbon matrix for sulfur materials to function as a current collector and electrolyte/polysulfides reservoir since the high electronic conductivity, structural high porosity, and mechanical stability. Polyaniline (PANI) is adopted to the S/SWNT composite to block the out-diffusion polysulfides while function as a gel electrolyte. Due to the electronic conductivity of PANI, it can also help to improve the composite conductivity, resulting in high sulfur utilization. The PANI coated S/SWNT composite (termed as PANI-S/SWNT) showed a superior specific capacity of 1011 mAh/g over 100 cycles and a good rate performance, indicating the synergic contribution of porous carbon and conducting polymer protection to alleviate challenges underlying sulfur cathode.

## Results and Discussion

**Figure 1:**
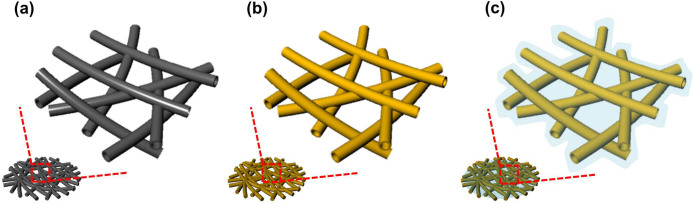
Schematic illustration of preparing the PANI-S/SWNT. (a) Freestanding SWNT film. (b) S/SWNT via sulfur thermo-infusion method. (c) Encapsulation of S/SWNT with PANI web.

Figure 1 shows the schematic illustration of preparing the PANI-S/SWNT composite. The freestanding SWNT film (Figure 1a) prepared by filtration method has a self-supported structure with high porosity, which can facilitate the electrolyte penetration and retain electrolyte as well as the dissolved polysulfides. The as-prepared SWNT film is then coated with sulfur by thermo-infusion method to get S/SWNT composite (Figure 1b). For the PANI coated S/SWNT, PANI is *in situ* synthesized on the S/SWNT surface to wrap and protect sulfur from being out-diffusion lost in liquid electrolyte (Figure 1c). Due to the PANI enhancement, the mechanically improved PANI-S/SWNT film can be directly used as sulfur cathode without following conventional electrode preparation process.

**Figure 2:**
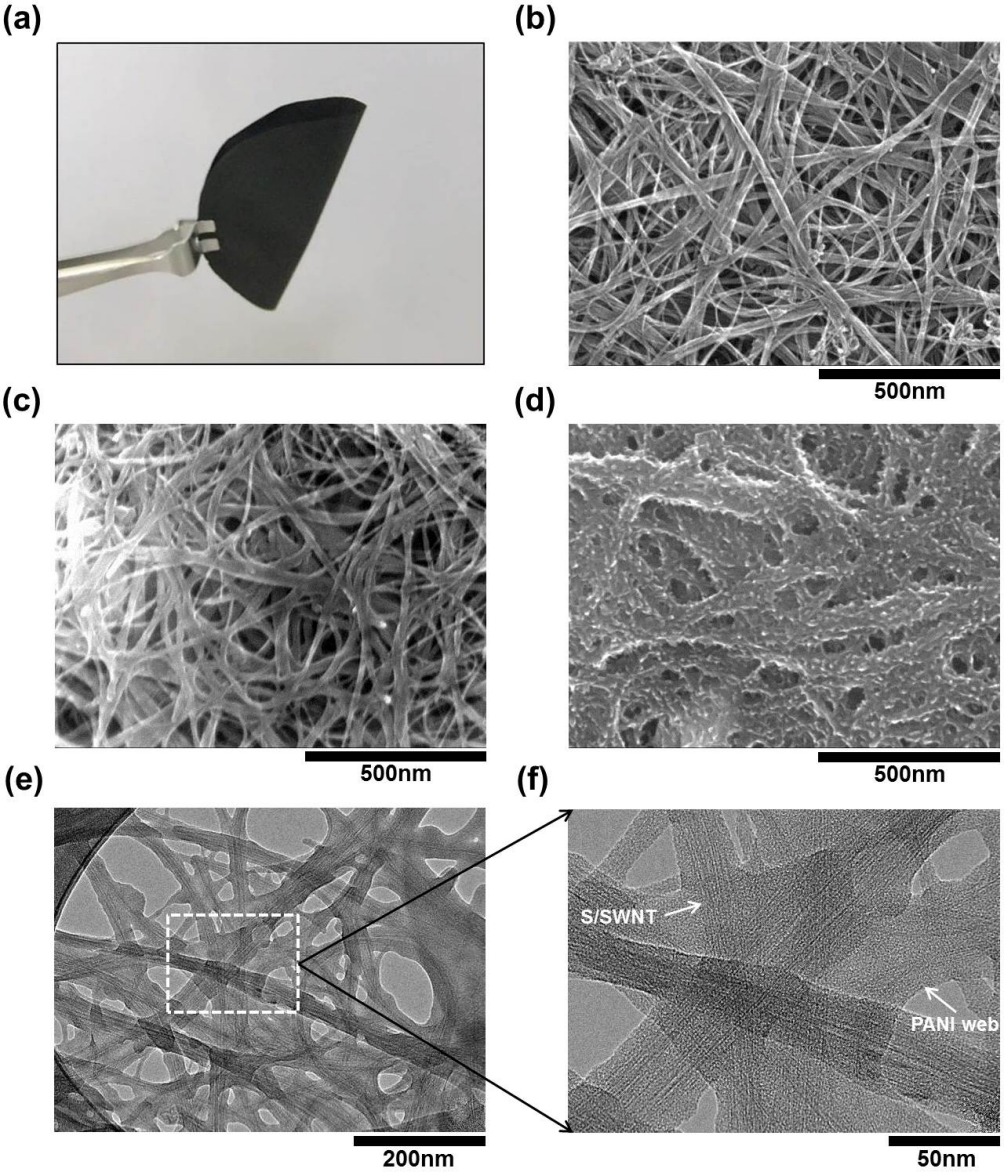
Morphological analysis. The photograph image of (a) folded S/SWNT film. The surface morphologies of (b) SWNT, (c) S/SWNT, (d) PANI-S/SWNT by SEM measurement. TEM images of PANI-S/SWNT in (e) low magnification and (f) high magnification.

Figures 2a shows a photo image of a folded S/SWNT film. In Figure 2b, the scanning electron microscopy (SEM) image of SWNT film shows a nonwoven structure having SWNTs self-supported with each other. After sulfur coating, the film remained stable and the increased diameter of SWNTs indicated the uniformly conformal sulfur coating (Figure 2c). To determine the sulfur distribution in the S/SWNT composite, energy-dispersive X-ray spectroscopy (EDS) mapping was conducted (Figure S1). The sharp contrast confirmed the heavy sulfur loading after sulfur thermo-infusion. For PANI-S/SWNT, rough surface was formed and the reduced charging effect in SEM image identified the enhanced conductivity by PANI coating (Figure 2d). To ascertain the thickness and the element distribution along the cross-sectional area of each S/SWNT and PANI-S/SWNT, cross-sectional EDS mapping analysis by SEM was conducted. (Figure S2 and S3) For further observation, transmission electron microscopy (TEM) was carried out to characterize the inner nanostructure of PANI-S/SWNT, showing that S/SWNT was fully encapsulated by amorphous PANI (Figure 2e–f). The PANI coating was around 20 nm thick. The EDS mapping in TEM confirmed the uniformly sulfur loading as well as PANI coating (Figure S4).

**Figure 3:**
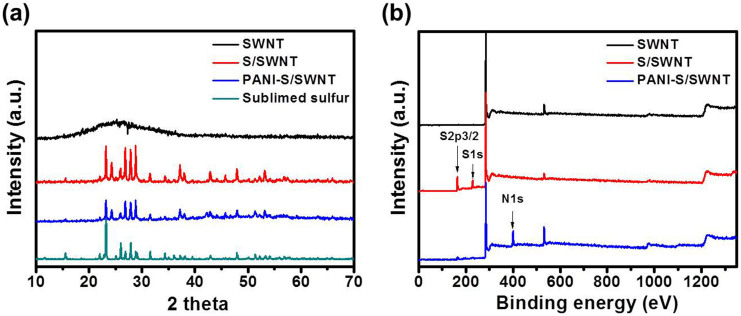
Characterizations. (a) XRD patterns of SWNT, S/SWNT, PANI-S/SWNT, and sublimed sulfur. (b) XPS spectra of S/SWNT and PANI-S/SWNT.

X-ray diffraction (XRD) patterns of SWNT, S/SWNT, PANI-S/SWNT, and sublimed sulfur are presented in the figure 3a. After sulfur coating, the S/SWNT showed the same patterns as the sublimed sulfur, indicating that sulfur was well distributed on SWNTs and the phase of sulfur was not changed after thermo-infusion treatment. For the PANI-S/SWNT, all the peaks followed the same positions as S/SWNT and sulfur except the decreased peak intensity. The XPS survey spectra of the S/SWNT and PANI-S/SWNT confirmed the PANI coating (Figure 3b). In the sample of S/SWNT, the peaks centered at 228.08 eV, 164.08 eV, 284.08 eV, and 532.08 eV were assigned to the S 1s, S 2p3/2, C 1s, and O 1s, respectively. The location of S 2p3/2 was consistent with the peak of aromatic S-C-S bond in sulfur-doped graphene, implying chemical bonding was formed between sulfur and SWNT in the sulfur thermo-infusion treatment^25^. For PANI-S/SWNT sample, N 1s peak centered at 400.08 eV demonstrated the PANI coating on S/SWNT based on the existing nitrogen in PANI polymer.

**Figure 4:**
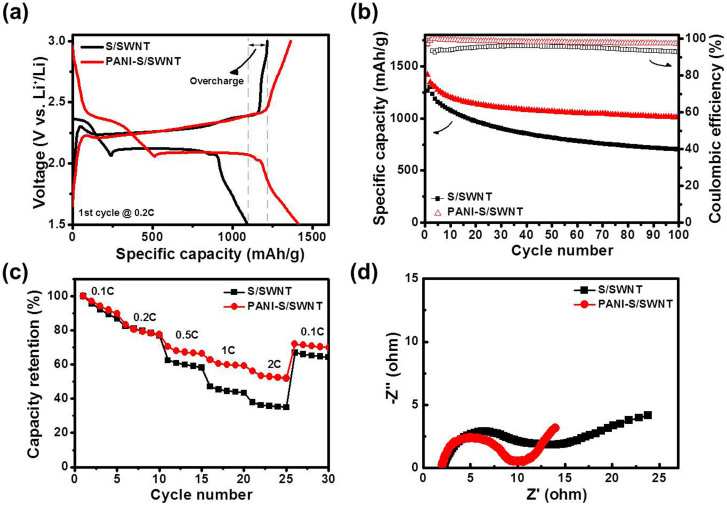
Electrochemical performances of S/SWNT and PANI-S/SWNT. (a) The initial discharge-charge curves, (b) Cycling performance, (c) Rate retention, (d) Nyquist plots of S/SWNT and PANI-S/SWNT electrodes.

The electrochemical performances of S/SWNT and PANI-S/SWNT are shown in Figure 4. Their galvanostatic discharge-charge curves at a current density of 0.2 C (1 C = 1672 mAh/g) are given in Figure 4a. The initial discharge and charge capacities of S/SWNT were 1096 mAh/g and 1217 mAh/g, respectively. The higher charge capacity was caused by polysulfides shuttle effect, indicating the migration of polysulfides between electrodes. The initial specific discharge-charge capacity of PANI-S/SWNT showed improved capacity of 1415 mAh/g and 1363 mAh/g, respectively, indicating the increased sulfur utilization and suppressed shuttle effect due to the conducting PANI coating. Considering the theoretical capacity of 1672 mAh/g, the sulfur utilization in S/SWNT and PANI-S/SWNT was 66% and 85%, respectively. After 100 cycles, the specific discharge capacity of S/SWNT decreased to 702 mAh/g, while PANI-S/SWNT retained 1016 mAh/g. The PANI-S/SWNT also showed a higher Coulombic efficiency of 98% than the S/SWNT (Figure 4b). The rate retention of S/SWNT and PANI-S/SWNT were measured by increasing the current density from 0.1 C to 2 C (Figure 4c). The rate retention was calculated by dividing each capacity with the numerical value of the first specific capacity at 0.1 C. At the current density from 0.1 C to 0.2 C, the trend of capacity fading showed similar behavior to the characteristic of rapid capacity fading over the first few cycles in lithium sulfur batteries. However, the rate retention of S/SWNT and PANI-S/SWNT showed remarkable difference as the current increases to a higher c-rate. At the current density of 2 C, S/SWNT exhibited a capacity retention as high as 37%, whereas PANI-S/SWNT showed a higher rate retention of 56%. The superior rate retention at a high current density can be mainly contributed to the conducting PANI coating on surface. The blanket-like PANI web structure provided an extra conducting pathway to the outer surface of sulfur, facilitating more and more sulfur activation. Moreover, the conformal PANI coating was able to confine the polysulfide locally and functioned as a protective shell to prevent electrolyte from directly contacting with sulfur, so as to inhibit the out-diffusion polysulfides and achieve excellent electrochemical performance. Electrochemical impedance spectroscopy (EIS) was also carried out to characterize the effect of PANI coating on the resistance of batteries. The EIS was surveyed in a frequency range of 250 kHz to 100 mHz with a scan rate of 5 mV and the Nyquist plots of S/SWNT and PANI-S/SWNT are shown in Figure 4d. The electrolyte resistance (intersection of the high-frequency portion of the semi-circle with the real axis) for the S/SWNT was 2.2 ohm while the PANI-S/SWNT was 2.0 ohm. The slightly decreased electrolyte resistance indicated that no polysulfides traveled out of the CNT structure, confirming the benefit of PANI protection on sulfur. The shortened length of the semi-circle demonstrated that conducting PANI coating on sulfur cathode can effectively decrease the electrochemical impedance of Li-S batteries.

**Figure 5:**
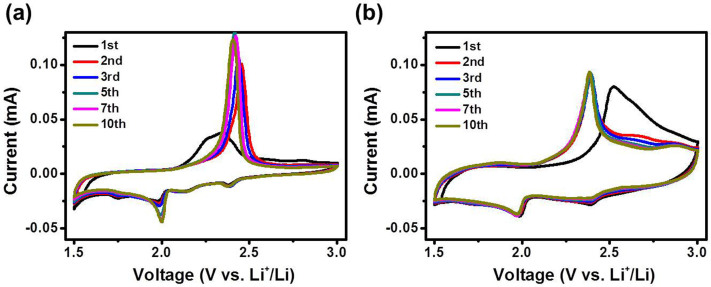
Cycle voltammetry curves of (a) S/SWNT and (b) PANI-S/SWNT during 10 cycles at the scan rate of 0.2 mV/s.

To analyze the electrochemical behavior of S/SWNT and PANI-S/SWNT, cyclic voltammetry (CV) profiles were collected during the initial 10 cycles at a scan rate of 0.2 mV/s in a voltage range of 1.5–3.0 V (V vs. Li/Li^+^). Typically, elemental sulfur features two reduction current peaks at ~2.3 V and ~2.0 V in liquid electrolyte, corresponding to a solid-to-liquid (from S_8_ to dissolved Li_2_S_8_) phase transition and a liquid-to-solid (from the dissolved Li_2_S_4_ to Li_2_S_2_/Li_2_S) phase transition, respectively^6^. In Figure 5a, CV curve of S/SWNT exhibited a typical reduction process of sulfur having reduction peak at 2.38 V and 1.98 V, respectively. An anodic peak at 2.45 V was the transformation of Li_2_S_4_ to high order of polysulfides. The CV curve of PANI-S/SWNT also showed a similar behavior of the electrochemical reaction with S/SWNT. However, the biggest difference between S/SWNT and PANI-S/SWNT is the reversibility of the electrochemical reaction during the cycles. The intensity and the position of anodic peak in S/SWNT electrode is negatively shifted after second cycle, implying that the sulfur reaction kinetics was not stable and electrode was still electrochemically activating during the repetitive cycles. In contrast, anodic peak in PANI-S/SWNT sample was repeatedly overlapped after second cycle, indicating that conducting PANI can stabilize the sulfur reaction kinetics and facilitate the electrochemical activation. In addition, the overall area of the scan window for PANI-S/SWNT is obviously larger than S/SWNT, showing that more sulfur was activated and utilized. The difference of peak potential between the lower plateau reaction of discharge and charge is denoted as ΔVp, which describes the sum of polarization upon the discharge and charge process. As shown in the Figure S5, ΔVp of PANI-S/SWNT showed a lower polarization value than S/SWNT, indicating that PANI-S/SWNT is more favorable for the reversible electrochemical reaction than S/SWNT.

In summary, we have developed a flexible S/SWNT film with conducting PANI coating as polysulfide block to increase the Li-S batteries electrochemical performance. The initial discharge capacity of PANI-S/SWNT reached 1415 mAh/g, which was higher than that of S/SWNT because that more sulfur was activated and utilized by the protection of conducting PANI coating. Moreover, the PANI-S/SWNT sample maintained its specific capacity of 1011 mAh/g at the 100^th^ cycle with a 20% higher capacity retention rate than that of S/SWNT. In particular, the rate capability of PANI-S/SWNT showed a higher capacity retention of 56% than that of S/SWNT (37%) at 2 C. The synergic contribution of porous carbon structure and conducting polymer protection provides an effective strategy to alleviate challenges underlying sulfur cathode. In addition, this facile and effective conducting polymer coating method can be extended to other conventional sulfur cathodes that require stable cycle stability and high capacity and other energy storage devices such as supercapacitor and Li-ion batteries.

## Methods

### Preparation of free-standing SWNT film

1 g of sodium dodecyl sulphate (SDS, ACS reagent, 99.0%, Sigma-Aldrich) was dissolved in 99 ml of de-ionized water (DI water) to prepare the 1 wt% of stock solution. 10 mg of SWNTs (Single-walled carbon nanotube, Cheaptube Inc.) were dispersed in 100 mL of SDS solution by using a direct tip sonication for 40 min to make a homogeneous solution. In order to remove any bundled SWNTs in the solution, the suspension was centrifuged at 2,000 rpm for 20 min and the resulting supernatant was filtered through a mixed cellulose acetate by using vacuum filtration system, followed by rinsing with 800 mL of DI water to remove the remaining SDS in the film. Finally, the SWNT on membrane film was dried in a fume hood for 2 hours to separate the freestanding film, followed by dipping in nitric acid for 30 min to completely remove the residual SDS.

### Synthesis of S/SWNT

Dried freestanding film was moved into the Teflon bottle containing 200 mg of sublimed sulfur. And then Teflon bottle was moved to the convection oven maintaining the temperature at 155 °C, and holding for 12 hours to coat the sulfur through the whole area of the SWNT film. After the coating process was finished, the bottle was slowly cooled to the room temperature.

Each weight of SWNT and sulfur in S/SWNT electrode is 0.28 mg and 0.61 mg, respectively. The ratio of sulfur to the S/SWNT is ~69%. It is well matched to the TGA curve. (Figure S6) After wrapping the S/SWNT with PANI web, the weight of it increases to 0.73 mg.

### Coating PANI onto S/SWNT film

100 μL of aniline (ACS reagent, ≥99.5%, Sigma-Aldrich) and 200 μL of concentrated hydrochloric acid (HCl, 36.5–38%, DAEJUNG CHEMICALS) were dropped into the 200 mL of bi-solvent composed of ethanol and DI water (1:1 = v:v), followed by stirring with a magnetic bar for 20 min to prepare homogeneous PANI coating solution. As-synthesized S/SWNT film was immersed into the as-prepared PANI coating solution and held for 15 min under magnetic stirring. Then 0.16 g of ammonium persulfate oxidant ((NH_4_)_2_S_2_O_8_) ACS reagent, ≥98.0%, Sigma-Aldrich) was added into the solution under stirring at room temperature. This process was maintained until the color of solution was changed from colorless to dark-blue. The PANI-S/SWNT was taken out of the solution, and then washed with enough DI water and ethanol to remove the precipitant of PANI on the film, followed by drying in the vacuum oven at 40 °C for 2 days.

### Fabrication and evaluation of the cell

The cell was fabricated with S/SWNT and PANI-S/SWNT as the cathode and lithium as the counter electrode. For the electrochemical characterization, the S/SWNT and PANI-S/SWNT were punched with a diameter of 0.375 inch. The electrochemical properties of S/SWNT and PANI-S/SWNT were evaluated in a binder free system using a coin-type half cell (2032R type). The loading mass of sulfur was calculated by subtracting the total weight before and after sulfur coating process, which was applied in evaluating the each cell of S/SWNT and PANI-S/SWNT electrodes. The electrolyte was 1 M lithium bis(trifluoromethanesulfonyl)imide (LiTFSI) in solvent mixture of 1,3-dioxolane and 1,2-dimethoxyethane (volume ratio 1:1) dissolved with 0.2 wt% of LiNO_3_. The role of LiNO_3_ is to form the passivation film on the surface of lithium metal, which can effectively suppress the redox shuttle of polysulfides to the lithium anode. The galvanostatic discharge-charge evaluations of the cell were done between 1.5 and 3 V vs. Li/Li^+^ using the battery cycle tester TOSCAT 3100 (Toyo Systems, Japan).

### Characterization methods

The morphologies of S/SWNT and PANI-S/SWNT were analyzed by using a JSM 4700F field emission scanning electron microscope (JEOL) and a JEM 2100F transmission electron microscope (JEOL). The percentage of sulfur loading weight in S/SWNT film was estimated by thermogravimetric analysis (TGA) with a Q500 thermogravimetric analyzer (TA Instruments) in the temperature range of 80 to 800°C at a ramping rate of 10°C/min in air. The crystallographic patterns of the SWNT, S/SWNT and sulfur were determined by using X-ray diffraction patterns using Cu-Kα radiation (Bruker Miller diffractometer). The electrochemical impedance spectroscopies (EIS) of S/SWNT and PANI-S/SWNT were evaluated using an Autolab PGSTAT 302N potentiostat/galvanostat apparatus (Metrohm AG) in the frequency range of 250 kHz to 100 mHz at an excitation amplitude of 5 mV. XPS analysis was carried out using a Sigma Probe (Thermo VG Scientific) with Al-Kα X-ray radiation.

## Author Contributions

J.K. and J.C. designed the study and carried out the experiments. K.K. and K.F. took the electrochemical measurement. X.H. performed the material characterization. J.K. drew the schematic illustration for the experimental idea. J.K., K.F., L.B. and U.P. drafted the manuscript. All the authors contributed to discussion and reviewed the manuscript.

## Supplementary Material

Supplementary Information Encapsulation of S/SWNT with PANI Web for Enhanced Rate and Cycle Performance in Lithium Sulfur Batteries
