# Supplementary material for: Encapsulation of S/SWNT with PANI Web for Enhanced Rate and Cycle Performance in Lithium Sulfur Batteries
Source: Sci Rep. 2015 Mar 10;5:8946. doi: 10.1038/srep08946 (PMC4354035; doi:10.1038/srep08946)
Supplement: Supplementary Information — Encapsulation of S/SWNT with PANI Web for Enhanced Rate and Cycle Performance in Lithium Sulfur Batteries [file srep08946-s1.pdf]

Supplementary Information

Encapsulation of S/SWNT with PANI Web for Enhanced Rate and Cycle Performance in Lithium Sulfur Batteries

Joo Hyun Kim[1], Kun Fu[3], Junghyun Choi[1,3], Kichun Kil[1], Jeonghyun Kim[2], Xiaogang Han[3], Liangbing Hu[3] & Ungyu Paik [1,2]

[1] *Department of Energy Engineering, Hanyang University, Seoul 133-791, South Korea*

[2] *Department of Materials Science and Engineering, Hanyang University, Seoul 133-791, South Korea*

[3] *Department of Materials Science and Engineering, University of Maryland, College Park, Maryland 20742, United States*

Corresponding authors.

Tel: +1-301-405-9303, E-mail: binghu@umd.edu (Prof. Liangbing Hu)

Tel: +82-2-2220-0502, E-mail: upaik@hanyang.ac.kr (Prof. Ungyu Paik)

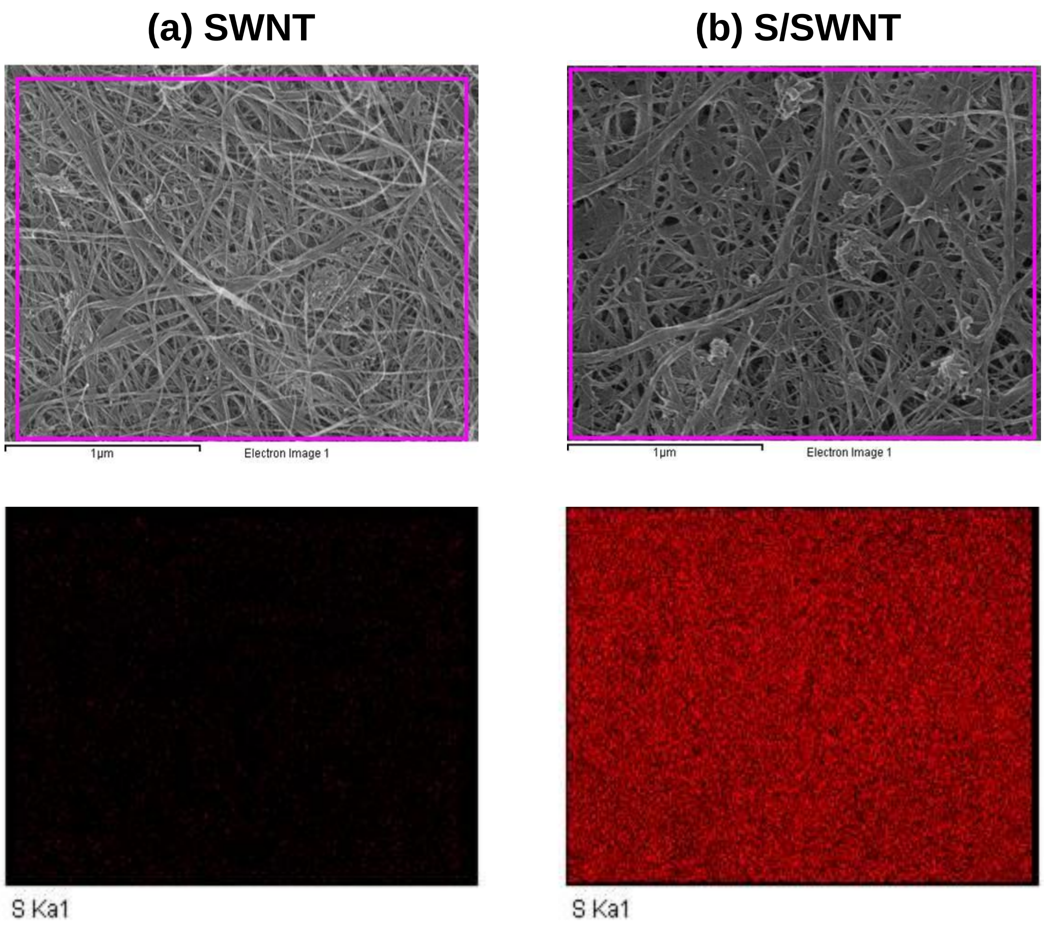

Figure S1 | EDS mapping images by SEM. (a) SWNT and (b) S/SWNT

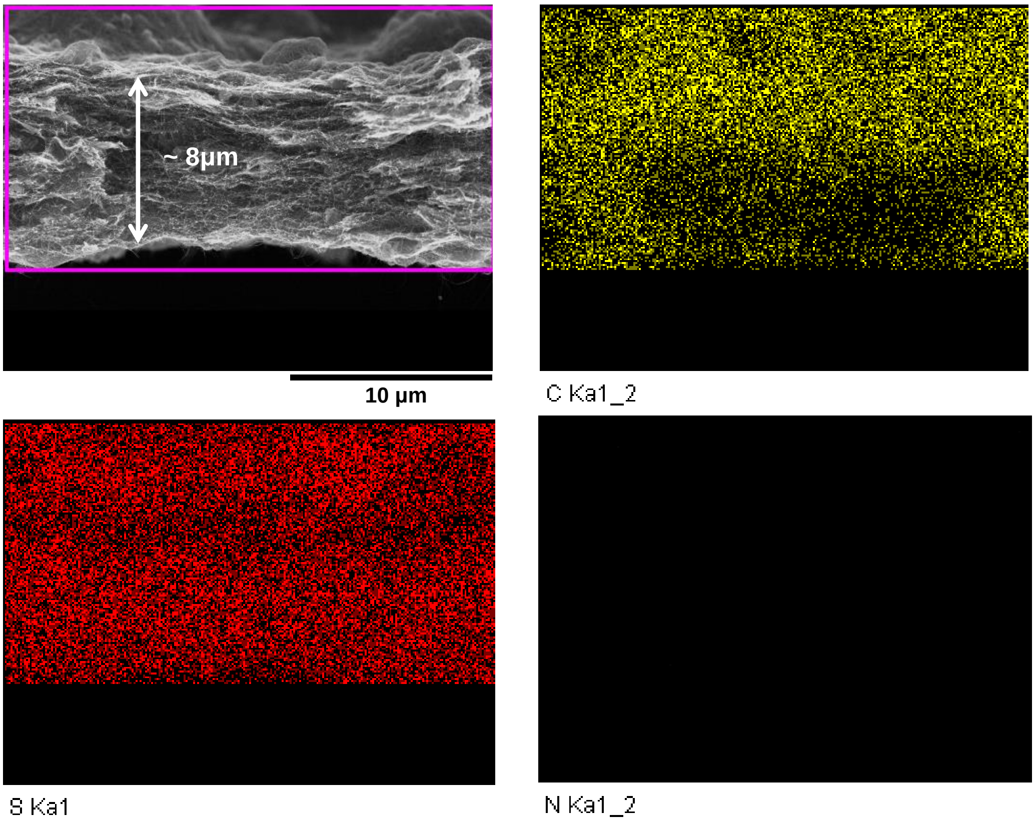

Figure S2 | Cross-sectional EDS mapping images of S/SWNT by SEM.

The thickness of the S/SWNT film is around ~8 μm. The element analysis by EDS mapping indicates the well-distributed sulfur element around the whole area of the cross-section.

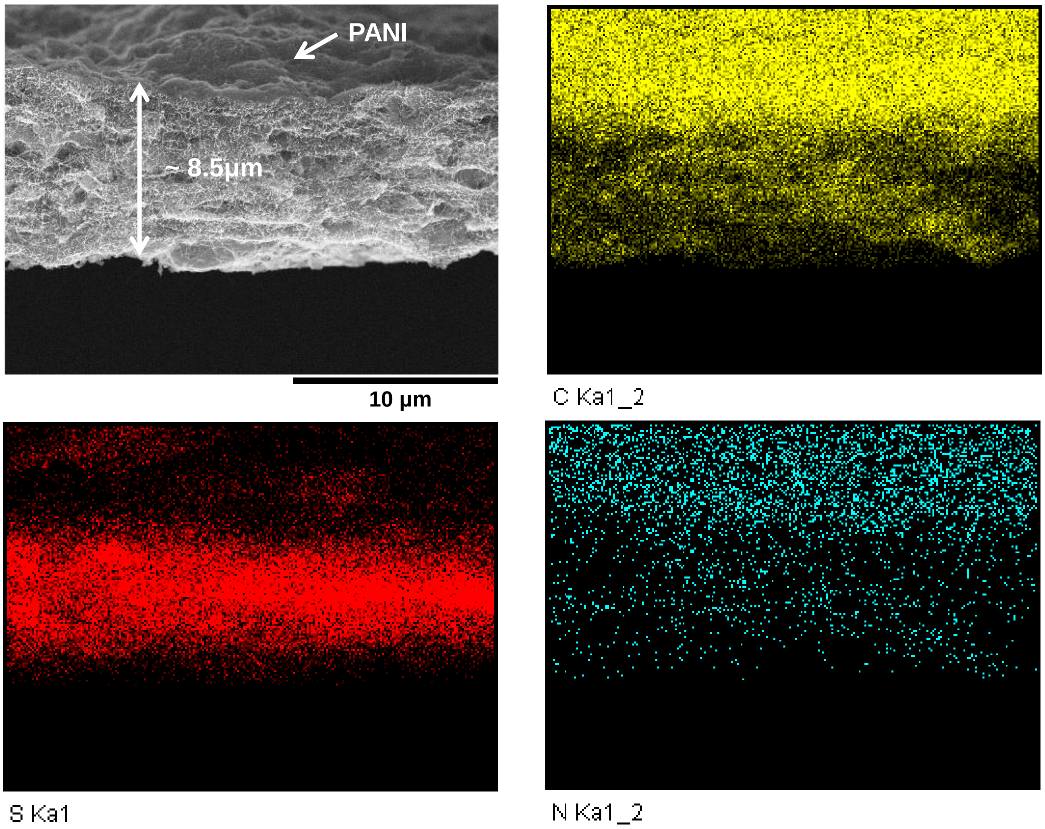

Figure S3 | Cross-sectional EDS mapping images of PANI-S/SWNT by SEM.

The thickness of the PANI-S/SWNT film is around ~8.5 μm. The element analysis by EDS mapping indicates the well-distributed sulfur around the whole area of the cross-section. Moreover, nitrogen element is only distributed on the surface of PANI-S/SWNT film, not inside of the film. This is attributed to the chemical element composing polyaniline.

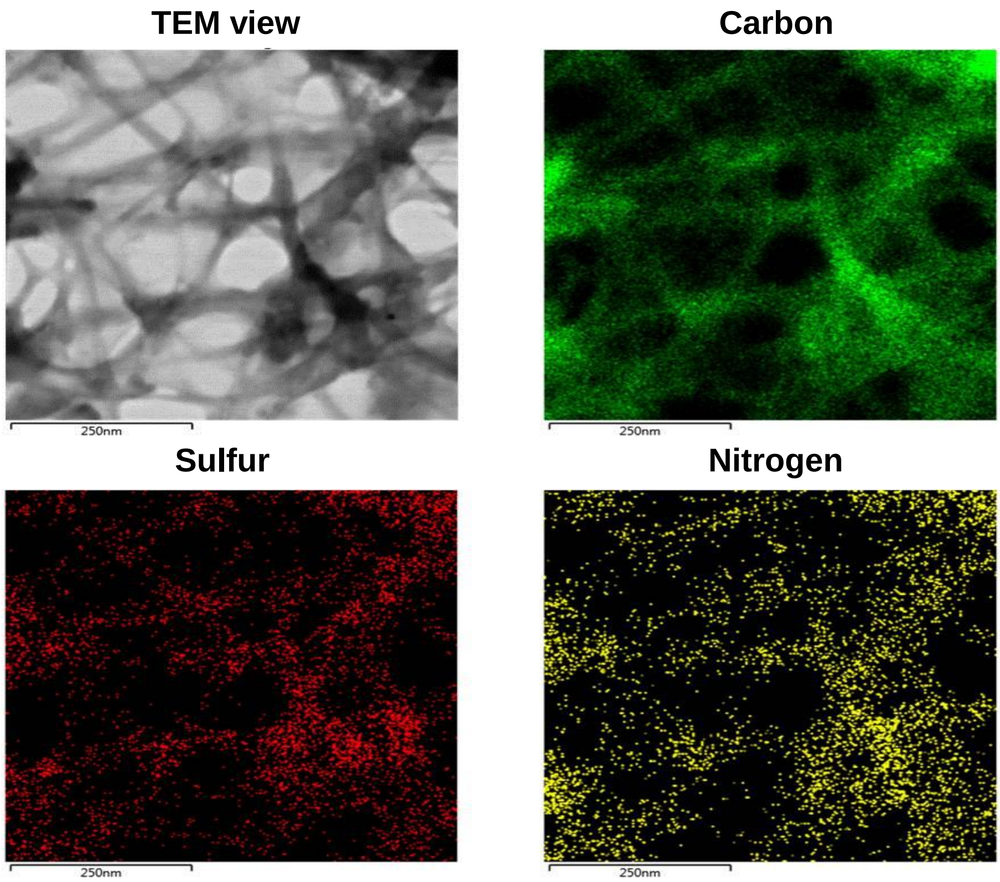

Figure S4 | EDS mapping images of PANI-S/SWNT by TEM.

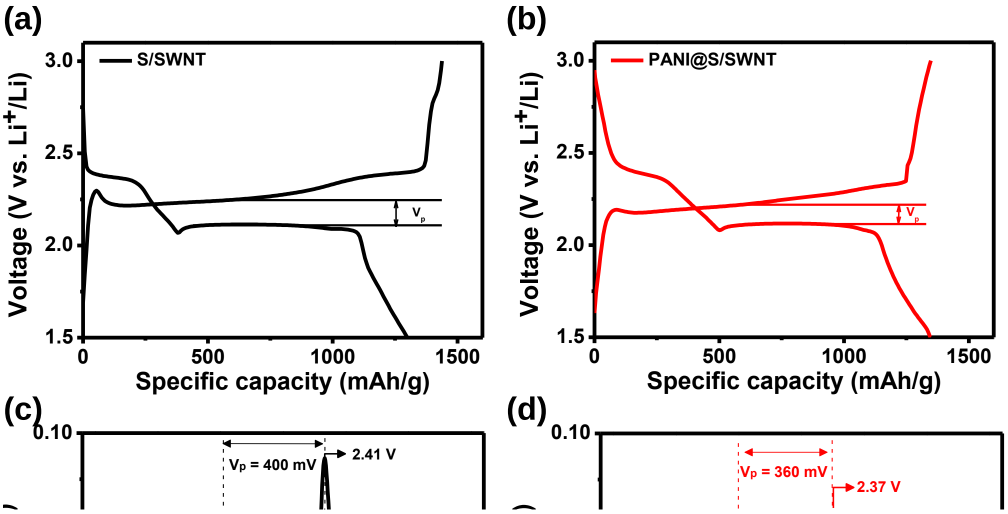

Figure S5 | Voltage discharge-charge curves of (a) S/SWNT and (b) PANI@S/SWNT at 2nd cycle. Cycle voltammetry curves of (c) S/SWNT and (d) PANI@S/SWNT at 2nd cycle

Figure S6 | TGA curve of S/SWNT sample between 40 ^{o}C and 800 ^{o}C.
